# Low Albumin Levels Are Associated with Poorer Outcomes in a Case Series of COVID-19 Patients in Spain: A Retrospective Cohort Study

**DOI:** 10.3390/microorganisms8081106

**Published:** 2020-07-24

**Authors:** Roberto de la Rica, Marcio Borges, Maria Aranda, Alberto del Castillo, Antonia Socias, Antoni Payeras, Gemma Rialp, Lorenzo Socias, Lluis Masmiquel, Marta Gonzalez-Freire

**Affiliations:** 1 Multidisciplinary Sepsis Group, Health Research Institute of the Balearic Islands (IdISBa), 07120 Palma de Mallorca, Spain; mborges@hsll.es (M.B.); maria.arandad@gmail.com (M.A.); alberto.delcastillo@gmail.com (A.d.C.); sociasmir@gmail.com (A.S.); 2 Intensive Care Unit, Son Llatzer University Hospital, Balearic Islands, 07198 Palma de Mallorca, Spain; grialp@hsll.es (G.R.); lsocias@hsll.es (L.S.); 3 Internal Medicine Unit, Son Llatzer University Hospital, Balearic Islands, 07120 Palma de Mallorca, Spain; apayeras@hsll.es; 4 Cardiopulmonary Pathology of the Critically Ill Patient Group, Health Research Institute of the Balearic Islands (IdISBa), 07120 Palma de Mallorca, Spain; 5 Vascular and Metabolic Pathologies Group, Health Research Institute of the Balearic Islands (IdISBa), 07120 Palma de Mallorca, Spain; Lluis.masmiquel@gmail.com

**Keywords:** COVID-19, albumin, inflammation

## Abstract

There is limited information available describing the clinical and epidemiological features of Spanish patients requiring hospitalization for coronavirus disease 2019 (COVID-19). In this observational study, we aimed to describe the clinical characteristics and epidemiological features of severe (non-ICU) and critically patients (ICU) with COVID-19 at triage, prior to hospitalization. Forty-eight patients (27 non-ICU and 21 ICU) with positive severe acute respiratory syndrome coronavirus 2 (SARS-CoV-2) infection were analyzed (mean age, 66 years, [range, 33–88 years]; 67% males). There were no differences in age or sex among groups. Initial symptoms included fever (100%), coughing (85%), dyspnea (76%), diarrhea (42%) and asthenia (21%). ICU patients had a higher prevalence of dyspnea compared to non-ICU patients (95% vs. 61%, *p* = 0.022). ICU-patients had lymphopenia as well as hypoalbuminemia. Lactate dehydrogenase (LDH), C-reactive protein (CRP), and procalcitonin were significantly higher in ICU patients compared to non-ICU (*p* < 0.001). Lower albumin levels were associated with poor prognosis measured as longer hospital length (r = −0.472, *p* < 0.001) and mortality (r = −0.424, *p* = 0.003). As of 28 April 2020, 10 patients (8 ICU and 2 non-ICU) have died (21% mortality), and while 100% of the non-ICU patients have been discharged, 33% of the ICU patients still remained hospitalized (5 in ICU and 2 had been transferred to ward). Critically ill patients with COVID-19 present lymphopenia, hypoalbuminemia and high levels of inflammation.

## 1. Introduction

The SARS-CoV-2 outbreak first identified in Wuhan in December 2019 has rapidly spread worldwide [1]. Spain has been hit particularly hard by the pandemic. By the time that this manuscript was written, more than 28,000 deaths related to COVID-19 have been confirmed. There is an urgent need to understand the causes behind these poor outcomes in order to improve patient management [1,2,3,4,5]. Thus, it is imperative to clinically characterize critically ill COVID-19 patients in order to identify those with a bad prognosis at an early stage, before their situation becomes irreversible.

COVID-19 has a rather heterogenous presentation. While many patients remain asymptomatic carriers, others can show a wide array of symptoms, from mild flu-like manifestations such as dry cough, phlegm, myalgia or diarrhea, to severe pneumonia or even acute respiratory distress syndrome (ARDS) [1,4]. The exact pathobiology responsible for severe and critically ill cases is still not clear. It has been proposed that a hyperinflammatory syndrome may play a central role in the progression from mild to severe or critical COVID-19 [6,7,8,9,10]. Inflammatory factors are likely involved in this process and could become biomarkers of disease progression in the near future [11,12]. Judging from similar hyperinflammatory syndromes like bacterial sepsis, fluctuations of these biomarkers will probably be strongly interrelated and time dependent [13]. Until this process is fully characterized, biochemical parameters and physical examinations are the only tools available for tracking disease progression. Efforts are being made in order to fully characterize the clinical characteristics of COVID-19 nationwide. The objective of this retrospective case series study was to describe the epidemiological and clinical characteristics of 48 hospitalized patients with COVID-19 and to compare patients who were admitted to the intensive care unit (ICU) care with those who did not receive ICU care, staying at a ward.

## 2. Materials and Methods

### 2.1. Study Population

The study was conducted at Son Llatzer University Hospital, a public tertiary care center covering a population of 280,000 from urban and rural areas in Mallorca, Balearic Islands, in Spain. The institutional review board approved this case series as minimal-risk research using data collected for routine clinical practice and waived the requirement for informed consent. According to the World Health Organization (WHO) guidance [14], laboratory confirmation for SARS-CoV-2 was defined as a positive result of real time reverse transcriptase–polymerase chain reaction (real time RT-PCR) assay from nasal and pharyngeal swabs (VIASURE SARS-CoV-2 S gene RT-PCR Kit CerTest Biotec, Zaragoza, Spain). Patients with confirmed SARS-CoV-2 infection by positive result on the real time RT-PCR test as of 31 March 2020, were admitted in the study. Fifty-two patients met the inclusion criteria, but of those, only 48 had epidemiological data. APACHE II Score, was calculated during the first 24 h after ICU admission to help determine the ICU patient’s mortality risk. Patients were admitted to Son Llatzer University Hospital between 15 March 2020, and 31 March 2020, inclusive of those dates. The patients were classified at the ICU according to the severity and the guidelines of the Son Llatzer University Hospital for COVID-19 management, as well as the criteria of the physicians. Clinical outcomes were monitored until the final date of follow-up. The follow-up data on clinical and laboratory measures are not included in this study.

### 2.2. Data Collection

Clinical and laboratory data were collected at triage by hospital staff (nurses). The data were recorded on electronic worksheets and uploaded to the health database. Three researchers independently reviewed the data collection forms for accuracy. Data collected included patient demographic information (age, sex, race, home medications, smoking habits), comorbidities, initial symptoms of the disease and triage vitals such as fever, oxygen saturation (SpO_2_), systolic and diastolic pressure and heart rate, as well as diagnosis of pneumonia by chest X-ray. In some instances, patients had missing data for the above parameters, in which case, percentages of the total patients with completed tests are shown. Initial laboratory testing was defined as the first test results available, typically within 24 h of admission. Complete blood cell count, tests of kidney and liver function and inflammatory and coagulation markers, such as C-reactive protein, lactate dehydrogenase, D-dimer, fibrinogen, troponin I and procalcitonin levels, were performed. Respiratory samples were tested for influenza and other respiratory viruses with a multiplex PCR assay. Patients underwent chest X-rays or computed tomography for pneumonia diagnosis. Supplemental oxygen was administered when saturations as measured by pulse oximeter dropped below 92%. Patients received antibiotics, anti-malaria drug (Hydroxychloroquine), co-formulated (“Kaletra”) antivirals lopinavir-ritonavir, immunosuppressive drugs such as Tocilizumab and Interferon beta and anti-inflammatory drugs. All patients received oxygen therapy.

### 2.3. Statistical Analysis

No statistical sample size calculation was performed a priori, and sample size was equal to the number of patients treated during the study period. Baseline characteristics of non-ICU versus ICU patients were summarized as means and standard deviations (SDs) for continuous variables and as frequencies and percentages for categorical variables. T tests were used to compare continuous characteristics and Fisher exact tests were used to compare categorical characteristics of non-ICU versus ICU patients when appropriate. Non-normal distributed continuous data were compared using the Mann–Whitney–Wilcoxon test. Log scale for some variables are presented when “physiological” outliers were detected. Differences in clinical and laboratory measures between non-ICU and ICU patients were assessed in multivariable linear regression models adjusted for age, sex, race, smoking status, SpO_2_ and data available on comorbidities (hypertension, dyslipidemia, type 2 diabetes, cardiovascular disease and COPD). Multivariable linear regression models were used to estimate the association between albumin levels and ICU-status with hospital length and mortality. Relationships were assessed using Spearman or Pearson correlations. All statistical tests were 2-tailed, and statistical significance was defined as *p* < 0.05. All analyses were performed using version 3.5.2 of the R programming language (R Project for Statistical Computing; R Foundation, Vienna, Austria. URL https://www.R-project.org/.).

### 2.4. Study Approval

The study was performed in accordance with Good Clinical Practice and the Declaration of Helsinki principles for ethical research. Ethical approval for this project (IB 4165/20 PI, 6 April 2020) was obtained from the ethics committee of the Balearic Islands. Written informed consent was waived due to the rapid emergence of this infectious disease.

## 3. Results

### 3.1. Clinical Features

**Figure 1:**
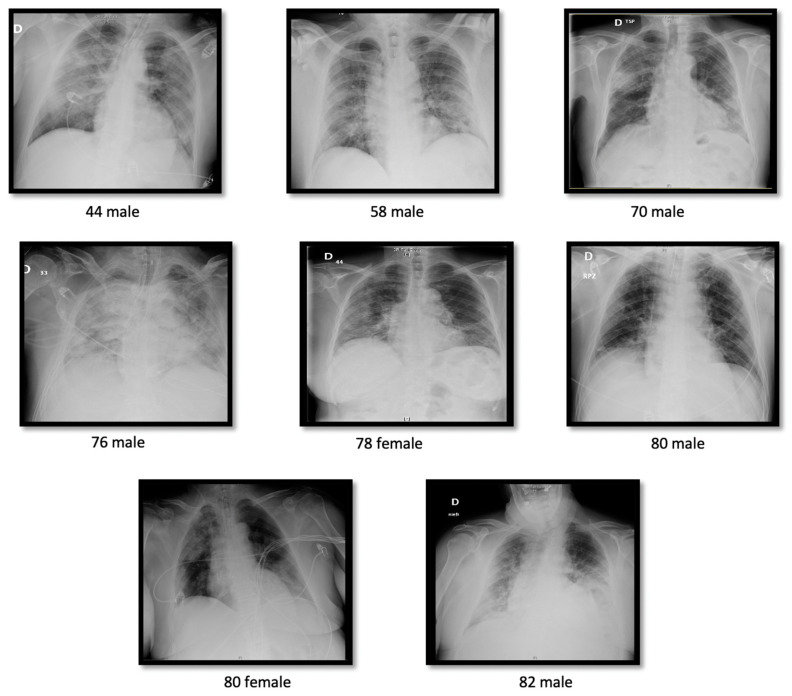
Chest X-ray images from all the deceased intensive care unit (ICU) patients. Most of the patients presented bilateral pneumonia at triage.

**Figure 2:**
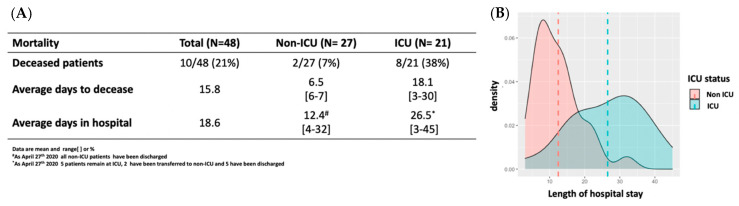
Mortality and length of hospital stay of the 48 COVID-19 patients. (**A**) Mortality, average days to decease and days in the hospital in non-ICU and ICU patients with COVID-19 in Mallorca. (**B**) Density plot of the length of hospital stay showing the average days in the hospital; red is non-ICU patients and green is ICU patients with COVID-19.

In this retrospective study, the clinical and epidemiological characteristics of 48 patients (mean age, 66 years, [range, 33–88 years]; 67% males) with laboratory confirmed COVID-19 by real time RT-PCR were analyzed. The patients were classified as non-ICU and ICU according to the severity and the guidelines of the Son Llatzer University Hospital for COVID-19 management. Table 1 shows the demographic, vital and clinical characteristics of the patients. There were no differences in age or sex among groups (*p* > 0.05). Comorbidities were identified in 70% of the patients, with hypertension (70%), dyslipidemia (62%) and cardiovascular disease (30%) being the most common. Initial symptoms included fever (100%), coughing (85%), dyspnea (76%), diarrhea (42%) and asthenia (21%). At triage, the average of SpO_2_ was 89%. ICU patients compared to non-ICU patients presented a significantly lower SpO_2_ (84% ± 12.51 vs. 93% ± 6.63, *p* < 0.001), and a higher prevalence of dyspnea (95% vs. 61%, *p* = 0.022) and ARDS (100% vs. 0%, *p* < 0.001). An abnormal chest radiograph presenting bilateral pneumonia was observed in 44 patients (94%) at admission. Only three non-ICU patients (11%) had unilateral pneumonia. Representative lung images showing interstitial lung abnormalities of the eight deceased UCI patients are presented in Figure 1. As of April 27, 2020, the overall mortality was 21% (10/48). Eight patients died in the ICU group (38%) vs. two in non-ICU (7%) (Figure 2). Treatments used to treat COVID-19 patients are summarized in Table S1. During hospitalization, 100% of the patients received Hydroxychloroquine; 98% received Kaletra, an antiviral treatment combining lopinavir and ritonavir; 100% received antibiotics; 54% corticosteroids and 100% received oxygen therapy. Tocilizumab was used in 48% of the ICU patients. We only had information on corticosteroids treatment in ICU. Due to missing information on renal treatment, those data are not presented. Invasive mechanical ventilation was received in all ICU patients. 

### 3.2. Laboratory Findings

#### 3.2.1. Hematologic Measures

Table 2 shows the hematologic measures at admission (mean (SD), range [ ] or %). Compared with the normal range, leukocyte or white blood cell (WBC) counts were normal in non-ICU vs. ICU patients, whereas lymphocyte counts were significantly lower in ICU patients (1.03 vs. 0.7 × 10^9^/L, respectively, *p* = 0.002), and below normal range ([1.00–4.5 × 10^9^/L]). Similarly, monocytes counts were significantly lower in the ICU group compared to non-ICU (0.58 vs. 0.40 × 10^9^/L, *p* = 0.029). After adjusting for possible confounders such as age, sex, race, smoking, SpO_2_, hypertension and dyslipidemia, lymphocyte and monocytes counts remained significantly lower in ICU patients (*p* = 0.045 and *p* = 0.040, respectively). When adding comorbidities such as type 2 diabetes, cardiovascular diseases and COPD into the model, the differences in monocyte and lymphocyte counts disappeared, although they were close to significant (*p* = 0.055 and *p* = 0.158, respectively). No differences in the number of red blood cells, hematocrit, hemoglobin, mean corpuscular volume (MCV), mean corpuscular hemoglobin (MCH), red blood cell distribution width (RDW), platelet distribution width (PDW), platelets and mean platelet volume (MVP) were found between non-ICU and ICU patients.

#### 3.2.2. Coagulation Function, Biochemical and Inflammation Measures

Table 3 shows the coagulation function, biochemical and inflammation measures admission (mean (SD), range [ ] or %). Due to the outliers in D-dimer and Ferritin, we show the data in these measures as mean (SD), range [ ], median (IQR) and in log scale. Prothrombin time was slightly higher in ICU compared to non-ICU patients (*p* = 0.038), but when adjusted for age, sex, race, smoking, SpO_2_, hypertension, dyslipidemia, type 2 diabetes, cardiovascular diseases and COPD, the difference disappeared. Mean levels of fibrinogen (713.63 mg/dL vs. 200–500 mg/dL) and median D-dimer (358 ng/mL vs. 0.00–255 ng/mL) were over normal range in COVID-19 patients. No differences were observed in fibrinogen or D-dimer levels among groups, but after adjusting for possible confounders, D-dimer levels were significant (*p* = 0.020).

All the biochemical measures were in normal range among groups except ferritin (572 ng/dL vs. 20–274 ng/dL), aspartate aminotransferase (AST) (44.5 U/L vs. 5.0–34.0 U/L), glucose (131.2 mg/dL vs. 70–100 mg/dL) and triglycerides (173.8 mg/dL vs. 0–150 mg/dL), where the overall mean was above the normal range.

**Figure 3:**
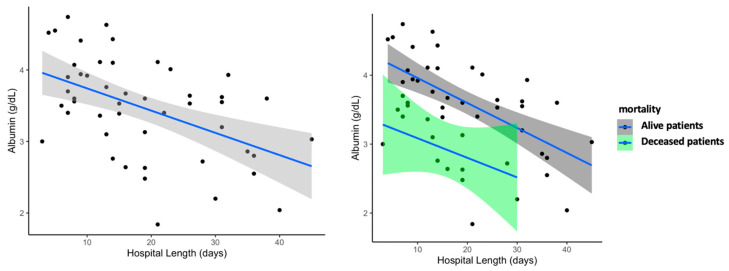
Association of serum albumin levels with hospital length in COVID-19. Patients with lower albumin stayed longer days in the hospital. On the right panel is a scatterplot showing the association of albumin levels and hospital day by groups; in green are the deceased patients (10 patients) and in grey are the alive patients (38 patients)**.**

No differences in creatinine, glomerular filtration rate, total bilirubin, alanine aminotransferase (ALT), phosphatase, gamma-glutamyl transferase (GGT), creatine kinase (CK), triglycerides, glucose or urea were found among groups. Urea was significantly lower in ICU patients when adjusted by age, sex, race, smoking, SpO_2_, hypertension, dyslipidemia, type 2 diabetes, cardiovascular diseases and COPD (*p* = 0.045). AST was significantly higher in ICU patients (*p* = 0.040), although after adjusting for all the confounders, the difference disappeared. Interestingly, albumin was significantly lower in ICU patients (*p* < 0.001), even in the adjusted models (*p* < 0.001). After this finding, we decided to study the association of albumin with the outcomes of prognosis of the disease. Overall, we found that lower albumin levels were associated with longer hospital length and mortality (r = –0.472, *p* < 0.001 and r = −0.424, *p* = 0.003, respectively) in COVID-19 patients (Figure 3). Table S2 shows the association between albumin levels and ICU-status with hospital length and mortality. Albumin levels were also positively correlated with the absolute number of lymphocytes (r = 0.368, *p* < 0.001), and negatively correlated with inflammatory markers such as procalcitonin (r = –0.555, *p* < 0.001), LDH (r = −0.443, *p* = 0.002), CRP (r = −0.390, *p* = 0.006), troponin I (r = −0.321, *p* = 0.026), ferritin (r = −0.506, *p* < 0.001) and MCV (r = −0.424, *p* = 0.003) among COVID-19 patients.

Interestingly, COVID-19 patients who died presented higher MCV compared to COVID-19 patients who survived (mean vs. 95.80 ± 3.32 vs. 89.79 ± 8.35 fl, respectively, *p* = 0.001) (data not shown).

**Figure 4:**
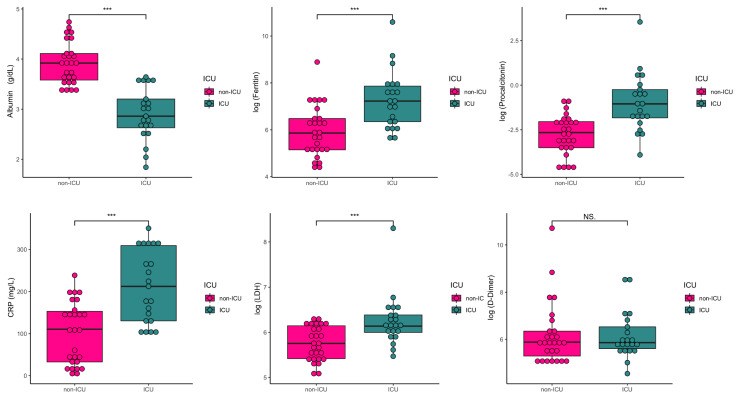
Representative scatterplots of inflammatory markers in the 48 patients with COVID-19. *** statistically significant; CRP (C-reactive protein); LDH (lactate dehydrogenase)

Finally, the mean of each inflammatory marker was in range among COVID-19 patients except lactate dehydrogenase (LDH) (479 vs. 124–243 U/L), troponin I (210.76 vs. 0.0–34.0 ng/L), C reactive protein (CRP) (150.43 vs. 0.0–5.0 mg/L) and procalcitonin (1.02 vs. 0.0–0.05 ng/mL), which were all higher. LDH, CRP and procalcitonin levels were significantly higher in ICU patients compared to non-ICU (*p* < 0.001). Troponin I levels became statistically different after adjusting for age, sex, race, smoking, SpO_2_, hypertension, dyslipidemia, type 2 diabetes, cardiovascular diseases and COPD (*p* = 0.045). Figure 4 shows the boxplots of the laboratory measures that presented more differences among non-ICU and ICU patients with COVID-19.

## 4. Discussion

To our knowledge, when this manuscript was submitted this study represented the first case series of sequentially hospitalized adult patients with confirmed COVID-19 in Spain. Knowledge of the baseline characteristics and outcomes of critically ill patients is crucial for health and government officials to address local outbreaks. Efforts are being made in order to fully characterize the clinical characteristics of COVID-19 nationwide. The overall mortality in this case series was 21%.

The majority of patients in this case series were hospitalized because of low SpO_2_ (89%), and presentation of bilateral pneumonia (94%). One-hundred percent of the ICU patients presented ARDS. These data are similar to those shown by similar studies from Italy and China. Most of the patients reported fever and a cough one week prior to hospitalization. Interestingly, among the 48 patients, 44% reported diarrhea as well, and this percentage was higher in the non-ICU group (44% vs. 31%, *p* = 0.070). These numbers are higher to those shown in previous reports (17–20%). Other common symptoms at onset of illness were fever, dry cough and dyspnea. One-hundred percent of the patients required oxygen therapy. All ICU patients required mechanical ventilation. ICU patients that died had a higher APACHE II score compared to ICU survivors (Figure S1).

Early publications from other countries show that epidemiological features such as age, sex and comorbidities play an important role in disease progression [15,16]. Men are more likely to show a poor prognosis than women [15]. Diabetes, chronic obstructive pulmonary disease (COPD) and hypertension are often comorbid in severe COVID-19 [16,17,18]. In our COVID-19 patients, hypertension was the most prevalent disease (70%), followed by dyslipidemia, cardiovascular disease and diabetes.

Overall, ICU patients presented lymphopenia and normal WBC, similar to other smaller case series reports of critically ill patients in the US, China, Singapore or Italy [1,19,20,21,22]. Patients who died, also presented lower lymphocytes compared to survivors (*p* < 0.0001, data not shown). Interestingly, MCV was also lower in deceased patients, although the numbers were in range (data not shown). We also found that critically patients had hypoalbuminemia, and that this remained statistically significant even after adjusting for possible confounders. To our knowledge, only two studies have reported similar data on albumin levels in COVID-19, but these results have not been emphasized [5,23]. Previous research has shown that hypoalbuminemia is a strong predictor of 30-day, all-cause mortality in critically ill patients [24]. In fact, in our case series, lower albumin levels were associated with mortality and length of hospital stay (Figure 3).

Serum albumin levels on admission predict the need of intensive respiratory support in adult patients with influenza A (H1N1) [25]. Lower levels of albumin have also been associated with higher inflammation, hypercoagulation and carotid atherosclerosis in people with human immunodeficiency virus infection (HIV) [26]. Low albumin levels have been associated with survival in COVID-19 patients as well [27].

Similar to other studies, ICU patients presented with inflammatory markers above the normal range, especially D-dimer, LDH, ferritin, fibrinogen, CRP, troponin I and procalcitonin. Contrary to other studies, the levels of D-dimer did not differ among groups. D-dimer levels were statistically significant only when the data were adjusted for sex, age, race, smoking, SpO_2_ and comorbidities. Higher levels of these inflammatory markers are indicative of coagulation, cardiac and renal dysfunction. In this study, hypoalbuminemia was also associated with inflammation, but little is known about the relationship between the prognosis and severity of the disease, inflammation and lower levels of albumin in COVID-19 patients. This relationship might be mediated by initial levels of inflammation. More studies are needed to corroborate this hypothesis.

Pro-inflammatory factors play a central role in COVID-19 severity, especially in patients with comorbidities [6,10]. The rapid virus replication rate along with interferon attenuation mechanisms and the accumulation of macrophages in the lungs seem to be important triggers of this “cytokine storm” [11]. We believe that it is imperative to measure inflammatory cytokines involved in the cytokine storm that has been seen in COVID-19. Polymorphisms in the angiotensin-converting enzyme receptor 2 (ACE2) are also likely to be involved in poor outcomes [28,29,30].

There is an urgent need to improve our understanding on the phenotype profiles behind the progress from mild to severe or critical COVID-19. To date, there is no specific treatment against COVID-19. In our cases series, 100% of the patients received a combination of antibiotic and antiviral treatments, and 81% received an antimalarial drug after being admitted to the hospital. Administering immunomodulators aimed to reduce COVID-19-driven inflammation comes with serious risks. Further studies are needed to characterize the effects of these drugs in cardiovascular, renal and pulmonary function in COVID-19 patients.

This study has several limitations. First, the study population is small and only includes patients from Son Llatzer hospital, one of the main hospitals within the Balearic Health system. There may be a selection bias when identifying factors that differ between non-ICU and ICU patients, even though the results were adjusted for known confounders, including age, sex, race, SpO_2_, smoking status and comorbidities. Also, this study is the first to show the characteristics of severe and critically ill COVID-19 adult patients in Spain. Secondly, this is a retrospective study done in an emergency situation. Third, the follow-up data could not be analyzed due to a delay in the update of the electronic health record database. Finally, some patients presented elevated biomarker data in some laboratory measures, and we did not exclude them from the analysis due to the small sample size and because those numbers were physiological and not due to technical error.

## 5. Conclusions

In conclusion, critically ill patients with COVID-19 present at triage with lymphopenia, hypoalbuminemia as well as high levels of inflammation. Lower levels of albumin were associated with poorer outcomes in COVID-19 patients. Albumin might be of importance because of its association with disease severity in patients infected with SARS-CoV-2. This small case series provides the first steps towards a comprehensive clinical characterization of severe and critical COVID-19 adult patients in Spain.

## Figures and Tables

**Table 1:** Baseline characteristics of 48 patients with COVID-19 at triage, prior to hospitalization.

Clinical Characteristics	All (*n* = 48)	Non-ICU (*n* = 27)	ICU (*n* = 21)	*p* Value
**At triage**				
**Age, yrs.**	65.98 (13.91) [33–88]	66.30 (14.90) [33–88]	65.57 (12.87) [44–82]	0.856
**Males, *n*%**	32 (67%)	18 (67%)	14 (67%)	1
**Fever, °C**	37.03 (0.94) [36–39]	36.84 (0.88) [36–39]	37.28 (0.98) [36–39]	0.147
**Systolic Pressure, mmHg**	129.6 (18.9) [90–180]	130.7 (16.6) [90–180]	128.1(21.9) [90–180]	0.642
**Diastolic Pressure, mmHg**	73.3 (11.7) [50–111]	75.9 (12.1) [50–110]	70.03 (10.44) [52–92]	0.058
**Heart Rate, bpm**	85.6 (14.59) [58–120]	86.9(17.4) [58–120]	83.8 (11.2) [58–106]	0.712
**Sp0_2_,%**	89.31(10.64) [38–99]	93.44(6.63) [66–99]	84(12.51) [38–99]	<0.001 *
**Symptoms Reported *n*%**				
**Asthenia**	10/46(21%)	6/25 (22%)	4/21 (19.1%)	1
**Dyspnea**	35/46 (76%)	16/26 (61%)	19/20 (95)	0.022 *
**Vomiting**	6/47 (13%)	4/25 (16%)	2/21 (9%)	0.870
**Diarrhea**	16/38 (42%)	12/25 (44%)	4/13 (31%)	0.070
**Coughing**	39/46 (85%)	20/27 (74%)	19/20 (95%)	0.225
**Fever**	48/48 (100%)	27/27 (100%)	21/21 (100%)	1
**ARDS**	20/46 (44%)	0/27 (0%)	21/21 (100%)	<0.001 *
**Pneumonia**	44/47 (94%)	24/27 (89%)	20/20 (100%)	0.078
**Bilateral pneumonia**	44/47 (94%)	21/27 (77%)	20/20 (100%)	NA
**Comorbidities *n*%**				
**Hypertension**	33/47 (70%)	22/27 (82%)	11/20 (55%)	0.101
**Dyslipidemia**	29/47 (62%)	16/27 (60%)	13/20 (65%)	1
**Type 2 Diabetes**	11/45 (24%)	9/27 (33%)	5/20 (25%)	0.286
**Cardiovascular disease**	14/47 (30%)	7/27 (26%)	7/20 (35%)	0.726
**Ictus**	3/46 (6%)	2/27 (7%)	1/20 (5%)	0.662
**Cancer or another malignancy**	10/47 (21%)	4/27 (15%)	6/20 (30%)	0.640
**COPD**	5/47 (11%)	4/27 (15%)	1/20 (5%)	0.544
**VIH**	1/46 (2%)	0/26 (0)	1/20 (5%)	0.894
**Renal chronic disease**	8/46 (17%)	4/27 (15%)	4/19 (21%)	0.877
**Other, *n*%**	26/47 (55%)	13/27 (52%)	12/20 (60%)	0.921
**Smoking**	10/47 (21%)	6/26 (22%)	4/20 (19%)	0.934

Data are mean (SD), range [] or %. * statistically significant. Not all the patients had complete information in of all the comorbidities, or symptoms. In those cases, the total number of patients are indicated for every outcome.

**Table 2:** Baseline laboratory measures of 48 Patients with COVID-19 at triage, prior to hospitalization.

	Normal Range	All (*n* = 48)	Non-ICU (*n* = 27)	ICU (*n* = 21)	*p* Value	*p* Value ^#^	*p* Value ^##^	*p* Value ^$^
**Leukocytes, 10^9^/L**	4.00–11.0	7.69 (3.40) [2.51–18.4]	7.48 (3.28) [3.76–18.4]	7.95 (3.61) [2.51–16.7]	0.582	0.987	0.897	0.345
**Neutrophil count, 10^9^/L**	1.80–7.50	6.11 (3.34) [1.66–16]	5.62 (3.12) [2.17–16]	6.76 (3.58) [1.66–15.7]	0.199	0.365	0.641	0.591
**Lymphocyte count, 10^9^/L**	1.00–4.5	1.03 (0.55) [0.29–2.82]	1.23 (0.57) [0.45–2.82]	0.77 (0.40) [0.29–2.03]	0.002 *	<0.001 *	0.045	0.158
**Monocyte count, 10^9^/L**	0.00–1.0	0.50 (0.29) [0.12–1.56]	0.58 (0.33) [0.12–1.56]	0.40 (0.20) [0.12–0.88]	0.029 *	0.032 *	0.040	0.055
**Red Blood cells, 10^12^/L**	4.50–5.8	4.45 (0.76) [2.35–6.8]	4.51 (0.68) [3.02–6.18]	4.37 (0.87) [2.35–6.8]	0.454	0.798	0.848	0.661
**Hemoglobin, g/dL**	13.00–16.7	13.29 (1.80) [7.66–16.3]	13.38 (1.70) [10.3–16.3]	13.16(19.96) [7.66–15.7]	0.795	0.730	0.640	0.277
**Hematocrit, %**	40.00–50.00	40.25(5.67) [22.5–50.3]	40.57(5.42) [30.6–50.3]	39.84(6.08) [22.5–50]	0.827	0.735	0.633	0.287
**Mean Corpuscular Volume (MCV), fl**	80.00–99.00	91.04 (7.94) [60.8–102]	90.16(7.91) [60.8–102]	92.17 (8.04) [63.9–101]	0.137	0.311	0.243	0.513
**Mean Corpuscular Hemoglobin (MCH), pg**	27.00–32.00	30.18 (2.91) [19.1–34.3]	29.93(2.96) [19.1–34.3]	30.49 (2.88) [20.3–33.7]	0.266	0.404	0.353	0.612
**Red Blood Cell Distribution Width (RDW), %**	10.00–14.00	12.57 (1.41) [10.90–19.8]	12.39(1.01) [10.9–15]	12.192(1.80) [11.40–19.8]	0.423	0.579	0.874	0.733
**Platelet Distribution width (PDW), %**	14.00–18.00	16.91 (1.13) [15–19.8]	16.81(1.24) [15–19.8]	16.99(0.97) [15.4–19.7]	0.333	0.930	0.960	0.864
**Platelets, 10^9^/L**	150.00–400.00	219.94 (96.04) [46.8–518]	228.02(108.82) [46.8–518]	209.56 (77.97) [81.7–429]	0.678	0.741	0.814	0.909
**Mean Platelet Volume (MPV), fl**	7.50–11.00	8.03 (1.22) [5.78–11.1]	7.92(1.25) [5.78–10.9]	8.18 (1.19) [6.71–11.1]	0.596	0.185	0.119	0.464

Data are mean (SD), range [ ] or %. * statistically significant. # *p*-value after adjustment by age, sex, race, smoking, hypertension and dyslipidemia. ## *p*-value after adjustment by age, sex, race, smoking, hypertension, dyslipidemia and SpO_2_. $ *p*-value after adjustment by age, sex, race, smoking, hypertension, dyslipidemia, SpO_2_, type 2 diabetes, cardiovascular disease and chronic obstructive pulmonary disease (COPD).

**Table 3:** Baseline coagulation function, biochemical and inflammation measures.

	Normal Range	All (*n* = 48)	Non-ICU (*n* = 27)	ICU (*n* = 21)	*p* Value	*p* Value ^#^	*p* Value ^$^
**Prothrombin time, s**	8.5–15.0	13.70 (1.59) [11.4–20.2]	13.20(1.12) [11.40–15.50]	14.33(1.89) [11.9–20.2]	0.038 *	0.614	0.461
**Fibrinogen, mg/dL**	200–500	713.63 (160.50) [405–1185]	686.44(151.77) [420–1118]	748.57 (168.27) [405–1185]	0.212	0.093	0.078
**D-Dimer, ng/mL ^&^**	0.00–255	1745.08 (6495.60)/358 (262–609) ^&^ [94–44,808]	2405.93(8583.03)/358 (201–2406) ^&^ [150–44,808]	895.43(1427.10)/350 (272–895^)&^ [94–5105]	0.731	0.279	0.020 *
**Log (D-Dimer)**		6.17(1.17) [4.5–10.7]	6.18(1.32) [5.0–10.7]	6.16(1.0) [4.5–8.5]	0.967	0.412	0.121
**Creatinine, mg/dL**	0.72–1.25	1.10 (0.83) [0.6–5.4]	1.09(0.68) [0.64–3.82]	1.11(1.01) [0.57–5.40]	0.739	0.842	0.405
**Glomerular Filtration Rate, mL/min**		77.17(25.44) [11–125]	76.15(25.99) [16–116]	78.48(25.28) [11–125]	0.830	0.852	0.883
**Ferritin, ng/mL**	20–274	2074.37 (5903)/572 (279–1401) ^&^ [76.9–40,000]	750.58(1374.38)/352(172–652)^&^ [76.9–7245.9]	3776.39(8603.70)/1373(571–2598)^&^ [261.8–40,000]	<0.001 *	0.054	0.084
**Log(Ferritin)**		6.51(1.32) [4.3–10.6]	5.93(1.09) [4.3–8.8]	7.26(1.24) [5.57–10.6]	<0.001	<0.001 *	0.008 *
**Total Bilirubin, mg/dL**	0.2–1.2	0.79 (0.45) [0.3–22]	0.70(0.28) [0.34–1.43]	0.91(0.58) [0.41–2.20]	0.339	0.399	0.387
**Albumin, g/dL**	3.50–5.50	3.47 (0.69) [1.8–4.7]	3.92(0.42) [3.36–4.74]	2.90(0.52) [1.84–3.64]	<0.001 *	<0.001 *	<0.001
**Aspartate aminotransferase (AST), U/L**	5.0–34.0	44.50(27.66) [3–125]	40.41(30.15) [3–118]	49.76(23.77) [22–125]	0.040 *	0.062	0.369
**Alanine aminotransferase (ALT), U/L**	1.0–55.0	39.75(30.28) [9–121]	40.67(35.23) [9–121]	38.57(23.19) [14–94]	0.323	0.367	0.665
**Phosphatase, U/L**	40–150	80.88(45.19) [25–250]	74.96(27.59) [34–143]	88.48(60.83) [25–250]	0.909	0.890	0.311
**Gamma-glutamyl transferase (GGT), U/L**	12.0–64.0	79.60(80.77) [11–433]	69.04(51.16) [13–186]	93.19(106.99) [11–433]	0.843	0.338	0.227
**Lactate dehydrogenase (LDH), U/L**	125–243	479.04(548.36) [156–4038]	335.82(123.6) [156–545]	663.19(789.6) [237–4038]	<0.001 *	0.005 *	0.113
**Creatine Kinase (CK), U/L**	30–200	181.79(298.45) [27–1909]	202.89(375.21) [29–1909]	154.67(157.89) [27–736]	0.473	0.321	0.265
**Log(CK)**		4.64(0.95) [3.3–7.5]	4.60(1.04) [3.37–7.55]	4.69(0.84) [3.3–6.0]	0.744	0.601	0.536
**Triglycerides, mg/dL**	0–150	173.79 (74.46) [64–399]	163.22(68.40) [64–388]	187.38(81.26) [79–399]	0.284	0.215	0.651
**Glucose, mg/dL**	70–110	131.23 (59.18) [80–431]	131.78(65.38) [80–431]	130.52(51.69) [89–337]	0.868	0.601	0.347
**Urea, mg/dL**	15–50	47.46 (38.64) [14–193]	51.04(40.51) [16–176]	42.86(36.55) [14–193]	0.442	0.423	0.045 *
**Troponin I, ng/L**	0.00–34.0	210.76(1094.37) [1.9–7572.6]	76.01(184.38) [1.90–723.1]	384.00(1647.5) [1.9–7572.6]	0.412	0.475	0.034 *
**C reactive protein (CRP), mg/L**	0.0–5.0	150.43(96.62) [0.5–350.4]	101.30(73.27) [0.5–238.9]	213.59(86.68) [98.80–350.4]	<0.001 *	0.011 *	0.028
**Procalcitonin, ng/mL**	0.00–0.05	1.02(4.98) [0.01–34.7]	0.10(0.11) [0.01–0.43]	2.21(7.47) [0.02–34.66]	<0.001 *	<0.001 *	0.016

Data are mean (SD), range [ ] or %. * statistically significant. # *p*-value after adjustment by age, sex, race, smoking, hypertension and dyslipidemia. $ *p*-value after adjustment by age, sex, race, smoking, hypertension, dyslipidemia, SpO_2_, type 2 diabetes, cardiovascular disease, COPD and median, which is also represented with range.

## Supplementary Materials

The following are available online at https://www.mdpi.com/2076-2607/8/8/1106/s1, Table S1: Treatments used in the 48 COVID-19 patients during hospitalization. Table S2: Coefficients from multivariable linear regression models estimating the association between albumin levels and ICU-status with hospital length and mortality (*n* = 48). Figure S1. Boxplot showing the differences in APACHE II Score, between alive vs. deceased ICU Patients (*n* = 13, alive ICU vs. *n* = 8, deceased ICU).
