# Supplementary material for: Low Albumin Levels Are Associated with Poorer Outcomes in a Case Series of COVID-19 Patients in Spain: A Retrospective Cohort Study
Source: Microorganisms. 2020 Jul 24;8(8):1106. doi: 10.3390/microorganisms8081106 (PMC7463882; doi:10.3390/microorganisms8081106)
Supplement: Supplementary file 1 [file microorganisms-08-01106-s001.pdf]

Supplemental Table 1. Treatments used in the 48 COVID-19 patients during hospitalization

COVID-19 treatment#	All (N= 48)	Non-ICU (N=27)	ICU (N= 21)
~~Chloroquine or~~ Hydroxychloroquine	47/47 (100%)	25/26 (96%) (chl)	19/21 (62%) (hyd)<br>2/21 (chl)
Remdesivir	2/47 (4%)	0/27	2/21 (9%)
Kaletra (lopinavir+ritovanir)	46/47 (98%)	25/26 (96%)	21/21 (100%)
Levofloxacin	4/47 (8%)	3/26 (11%)	1/21 (5%)
Piperacillin+Tazobactam	2/47 (4%)	0/27	2/21 (9%)
Meropenem	4/47 (8%)	1/26 (4%)	3/21 (14%)
Ceftriaxone/+ Azithromycin	26/47 (55%)	16/26 (62%)	10/21 (48%)
Azithromycin	16/21 (76%)	0/27	16/21 (76%)
Cefotaxime /+ Azithromycin	14/47 (45%)	10/26 (38%)	4/21 (19%)
Tocilizumab	11/47 (23%)	1/26 (4%)	10/21 (48%)
Interferon beta	10/21 (48%)	0/27 (0%)	10/21 (48%)
Corticosteroids	26/48 (54%)	14/27 (52%)	12/21 (57%)
Oxygen Therapy	46/46 (100%)	25/25 (100%)	21/21 (100%)
Invasive Mechanical Ventilation	21/48	0/27	21/21 (100%)

Data are N and %
# p value not applicable

Supplemental Table 2. Coefficients from multivariable linear regression models estimating the association between albumin levels and ICU-status with hospital length and mortality (n = 48).

Coefficient	Albumin levels (N= 48)			
	Hospital length#		Mortality$	
	β (se)	p	β (se)	p
(Intercept)	4.50 (0.310	<.001	4.47 (0.31)	<.001
ICU status	**-0.93 (0.15)**	**<.001**	**-1.01 (0.13)**	**<.001**
Age	-0.01 (0.01)	.053	-0.01(0.01)	.079
Male	0.23 (0.12)	.063	0.24 (0.12)	.061
Race	0.52 (0.16)	.003	0.50 (0.16)	.005
Smoking	-0.31 (0.14)	.034	-0.28 (0.14)	.055
Hypertension	-0.27(0.17)	.135	-0.25 (0.17)	.168
Dyslipidemia	-0.02 (0.12)	.885	-0.04 (0.13)	0.743

adjusted R squared: 0.704, p <.001; $ adjusted R squared: 0.697, p <.001

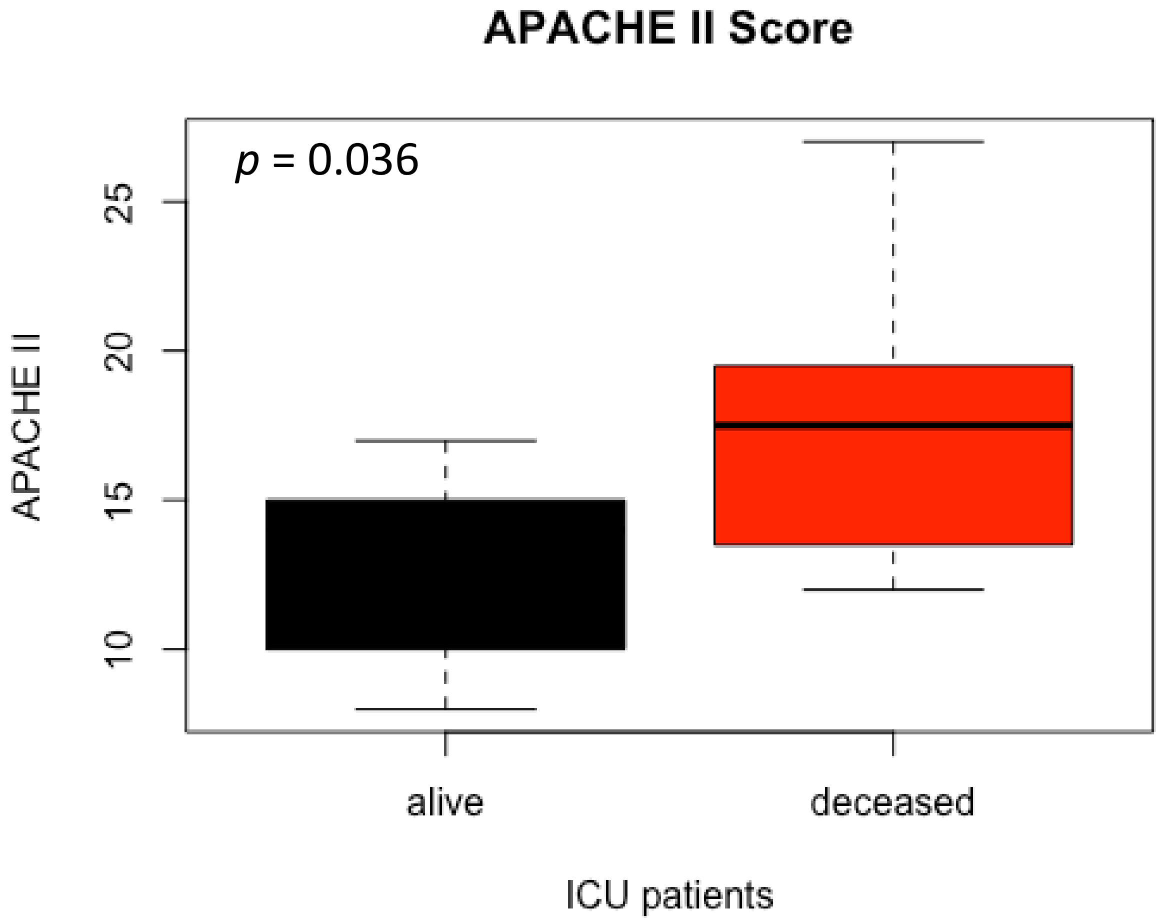

Supplemental Figure 1. Boxplot showing the differences in APACHE II Score, between alive vs deceased ICU Patients (N= 13, alive ICU vs N= 8, deceased ICU)
